# Exploring the distribution of grey and white matter brain volumes in extremely preterm children, using magnetic resonance imaging at term age and at 10 years of age

**DOI:** 10.1371/journal.pone.0259717

**Published:** 2021-11-05

**Authors:** Hedvig Kvanta, Jenny Bolk, Marika Strindberg, Carmen Jiménez-Espinoza, Lina Broström, Nelly Padilla, Ulrika Ådén

**Affiliations:** 1 Department of Women’s and Children’s Health, Karolinska Institute, Stockholm, Sweden; 2 Clinical Epidemiology Division, Department of Medicine, Solna, Karolinska Institutet, Stockholm, Sweden; 3 Department of Clinical Science and Education Södersjukhuset, Karolinska Institutet, Stockholm, Sweden; 4 Sachs’ Children and Youth Hospital, South General Hospital, Stockholm, Sweden; 5 Faculty of Health Sciences, Department of Basic Medical Sciences, Physiology Section, University of La Laguna, Tenerife, Spain; 6 Department of Neonatology, Karolinska University Hospital, Stockholm, Sweden; Western University, CANADA

## Abstract

**Objectives:**

To investigate differences in brain volumes between children born extremely preterm and term born controls at term age and at 10 years of age.

**Study design:**

Children born extremely preterm (EPT), up to 26 weeks and 6 days gestational age, in Stockholm between January 1 2004 to March 31 2007 were included in this population-based cohort study. A total of 45 EPT infants were included at term age and 51 EPT children were included at 10 years of age. There were 27 EPT children included at both time points. Two different control groups were recruited; 15 control infants were included at term age and 38 control children at 10 years of age. The primary outcomes were the grey and white matter volumes. Linear regression, adjusted for intracranial volume and sex, was used.

**Results:**

At term age, the extremely preterm infants had significantly smaller grey matter volume compared to the control infants with an adjusted mean difference of 5.0 cm^3^ and a 95% confidence interval of −8.4 to −1.5 (*p* = 0.004). At 10 years of age the extremely preterm children had significantly smaller white matter volume compared to the control children with an adjusted mean difference of 6.0 cm^3^ and a 95% confidence interval of −10.9 to −1.0 (*p* = 0.010).

**Conclusion:**

Extremely preterm birth was associated with reduced grey matter volume at term age and reduced white matter volume at 10 years of age compared to term born controls.

## Introduction

As neonatal care advances, more children who are born extremely preterm (EPT) survive [1, 2]. The Extremely preterm Infants in Sweden Study (EXPRESS) focuses on all children born alive up to gestational age (GA) 26 weeks and 6 days in Sweden between April 1 2004 to March 31 2007, and has expanded our understanding of the challenges faced by this vulnerable group [3]. Sweden has reported high survival rates for EPT children [4], and they have increased over time with more proactive care [5].

Extremely preterm birth affects the brain in numerous and complex ways and the mechanisms are yet to be fully elucidated [6]. In Sweden, one third of the EPT children included in the EXPRESS study had moderate or major cognitive impairments at 6.5 years of age [7]. Cognitive follow-up programs from other countries have reported similar risks of cognitive impairments [8, 9]. Also one third of EPT children in EXPRESS who were without major impairments had developmental coordination disorder at age 6.5 years [10].

Magnetic resonance imaging (MRI) has made it possible to further understand the brain development of preterm children. The majority of previous studies have focused on very preterm infants, defined as GA up to 31 weeks and 6 days, and have reported smaller global and regional brain volumes at term age compared to term born controls [11–13]. Cross-sectional studies of very preterm children have demonstrated that the smaller global brain volumes remain during childhood and adolescence [14, 15]. On a regional level, most brain regions in very preterm children show smaller volumes than term born controls when they become adolescents and young adults [16–19]. Some regions have been reported to be larger in the very preterm born groups than in term born controls in adolescence and adulthood, especially in higher order cognitive areas in the frontal and parieto-temporal cortex [17–19]. One previous longitudinal study of very preterm children showed growth reductions mainly in grey matter (GM) volume, from term age up to 7 years of age [20]. Studies that have compared preterm and term born children have reported greater reductions in brain volumes with lower gestational ages at birth at various ages [11, 12, 21, 22]. Children born at the lowest gestational ages are also more likely to have other morbidities and cognitive impairments [7, 9]. Few studies investigated EPT children, and at term age smaller global and regional brain volumes for EPT children than for term born controls have been reported, but our group also found that EPT infants had some larger brain regions compared to term born controls, mainly in the regions involved in visual processing [13, 22]. One study of only EPT children scanned with MRI at age 18 also reported smaller global brain volumes than term born controls, however this cohort was born in the early 1990s, and the caring of EPT children has since developed [23]. A few studies have shown that smaller GM and white matter (WM) volumes correlated positively with lower cognitive function [16, 20, 24–28].

However, the literature exploring global brain volume development of EPT children, born at the border of viability, is scarce. Cohorts previously investigated include children with a higher mean birth weight or include more sick children with a higher ratio of perinatal complication [12, 16, 20, 23]. We are not aware of other studies examining exclusively children born before 26 weeks and 6 days of gestation with MRI scans at term age and at 10 years of age.

Our aim in this study was to investigate the global brain volume development in a cohort born before 26 weeks and 6 days, compared to term born controls at term age and at 10 years of age.

## Materials and methods

### Study population

This population-based cohort study of EPT children is partly overlapping with the EXPRESS study [3]. The definition of EPT was the same as in EXPRESS–birth up to GA 26 weeks and 6 days.

**Fig 1:**
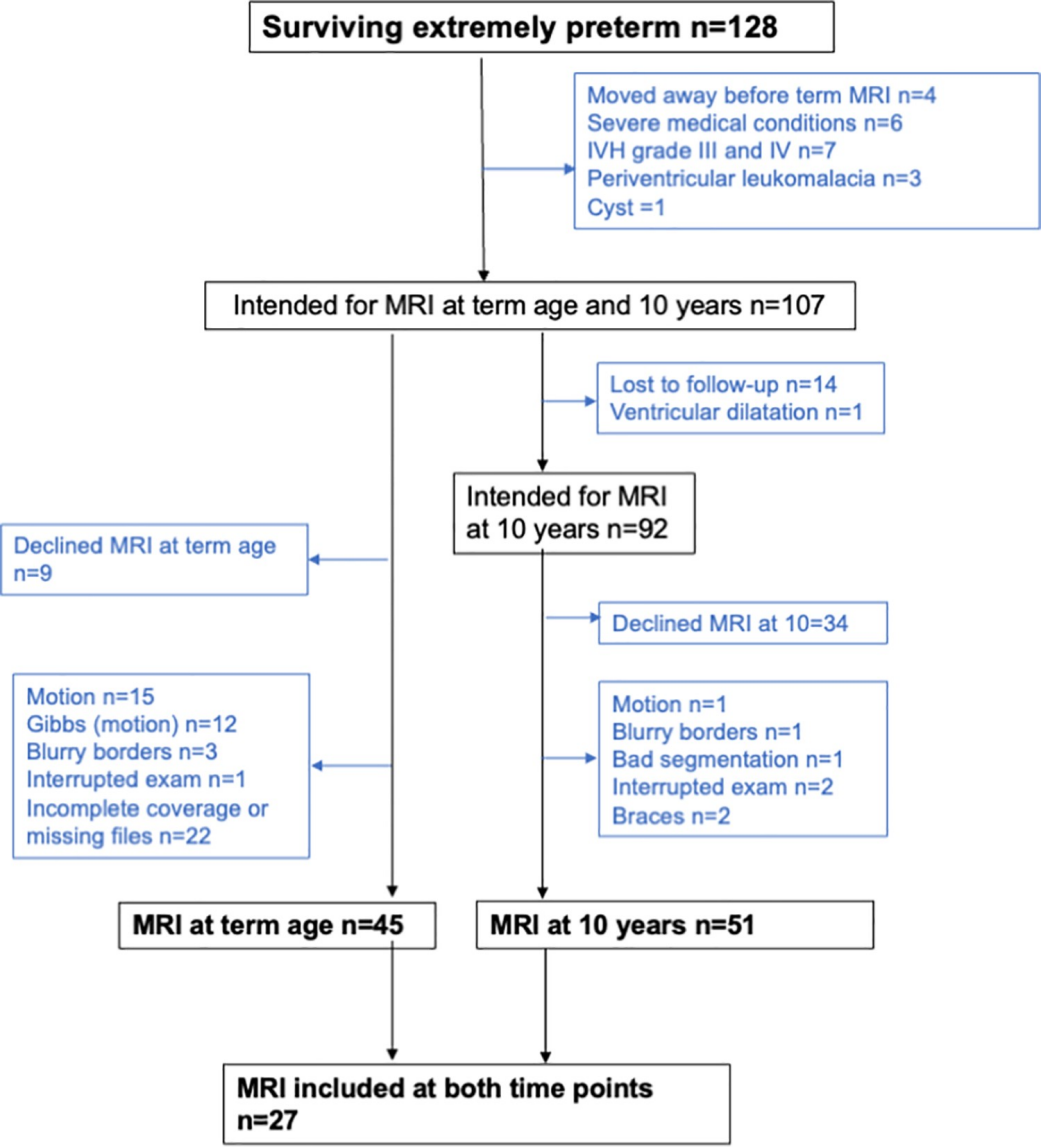
Flow chart for extremely preterm children. Extremely preterm children alive at birth, in Stockholm from January 1 2004 to March 31 2007 who underwent magnetic resonance imaging (MRI) scans at term age and at 10 years of age.

The study population is presented in Fig 1. We included all live born EPT children in Stockholm that were born between January 1 2004 to March 31 2007 (n = 191). There were 128 (67.0%) infants who survived to term age (GA 40 weeks and 0 days).

The exclusion criteria were lack of parental consent, severe medical conditions, major brain lesions (cystic periventricular leukomalacia, intraventricular haemorrhage (IVH) grade 3 and 4 diagnosed with cranial ultrasound during the neonatal period, focal brain lesions, cysts and severe white matter abnormalities on MRI, as defined by a previously published scoring system [29]) and low quality on MRI (defined as incomplete coverage of the brain, motion artifacts or blurring of the gray and white matter interfaces). Six infants were excluded for severe medical conditions; one with hemophagocytic lymphohistiocytosis, one too unstable to undergo MRI, three due to congenital malformations and one due to chromosomal abnormalities (trisomy 21). After exclusions, high quality MRI data (defined as images with complete coverage of the brain, free of motion artifacts, well defined contrast between GM, WM and CSF (cerebrospinal fluid), as well as legibility of anatomical structures) at term age (median age 40.7, range 39.1–45.3, weeks) were available for 45 EPT infants. One mother had more than one child included at term age.

At approximately 10 years of age the EPT children were invited for a follow-up MRI scan. The exclusion criteria were the same as at term age, but we did not exclude children who were not scanned at term age. Thus, there were 51 EPT children included with high quality MRI data at a median age of 10.3 years (range 9.0–11.8 years) (Fig 1). Three mothers had more than one child included at 10 years of age. There were 27 EPT children with high quality MRI data included at both term age and at 10 years of age. At 12 years of age the children had their anthropometric measurements (height, weight and head circumference) taken, and 34 EPT children had available anthropometric measurements at 12 years of age.

At term age 21 term born infants were recruited from the maternity ward as control infants. They were all healthy and had been delivered by elective caesarean sections and underwent MRI at a median age of 40.4 (39.9–41.9) gestational weeks according to the same protocol as the EPT infants. There were 15 control infants included with high quality MRI data at term age (S1 Fig).

At two years of age healthy, singleton full term children were randomly selected from the Swedish Medical Birth Registry and matched to the EPT children for place of birth, sex, day of birth and maternal country of birth. These children were used as control children at 10 years of age and were not significantly different from the term age controls with regard to sex and birth weight. The control children underwent MRI at a median age of 10.1 (range 8.3–11.6) years. Twelve of the control children were excluded, leaving 38 control children included at 10 years of age (S1 Fig). The same control children were approached for anthropometric measurements (height, weight and head circumference) at 12 years of age and 25 control children were included. The Stockholm ethics review board approved the study, and written, informed consent was obtained from the parents.

### Baseline characteristics

Perinatal data were retrieved from medical records. The Z-scores for weight and height were calculated from the reference material used in clinical practice in Sweden [30]. Sepsis was defined as either a positive blood culture or clinical symptoms of sepsis in association with an elevated C-reactive protein or leukocyte count. Small for gestational age was defined as a birth weight of more than 2 standard deviations (SD) below the mean for GA. Necrotizing enterocolitis was defined according to the Bell criteria [31]. Bronchopulmonary dysplasia was defined as the need for supplementary oxygen at 36 weeks of gestation.

### MRI data acquisition

At term age, imaging was performed on a Philips Intera 1.5-T MRI system (Philips International, Amsterdam, The Netherlands) and details of the sequence parameters have previously been published [32]. During the early study period the infants were given a low dose of chloral hydrate (30 mg/kg) given orally or rectally before the MRI examination, but during the last year of the study, most infants were scanned during natural sleep [32]. During the last year of the study infants were scanned during natural sleep. The imaging at 10 years of age, was performed on a General Electric 3.0-T MRI system (GE Healthcare, Milwaukee, WI, USA) and all the children underwent the MRI scan without sedation. The MRI protocol included a sagittal 3-dimension T1-weighted image with a BRAVO SPRGR sequence of 400 milliseconds, a field of view of 240x 240 mm^2^, a flip angle of 12^o^, a voxel size of 1 x 0.938 x 0.938 mm^3^ and a slice thickness of 1.0 mm. All the structural scans were assessed by a neuroradiologist.

### Brain segmentation and brain volumetry

**Fig 2:**
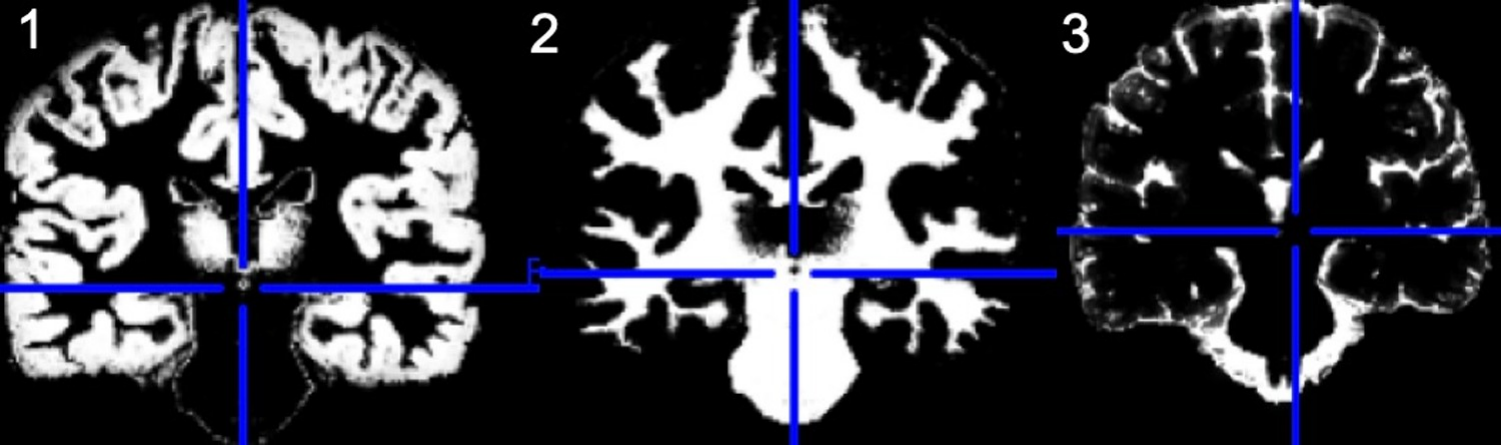
Brain segmentation. Coronal view of T1 weighted image segmented into 1) grey matter, 2) white matter and 3) cerebrospinal fluid.

The imaging processing for volumetry at term age has been previously published [22]. Our group has previously published brain volume measurements for cortical GM, cerebellum, WM, deep GM, CSF and brainstem at term age for part of this cohort [22]. The preparation of the 3-dimension MRI images at 10 years of age comprised of two stages. The first was reorientation of the T1-weighted images in the plane of anterior posterior commissures. The second was the removal of non-brain tissue components using the Brain Extraction Tool from the FMRIB Software Libraries version 5.0.1 (FMRIB Laboratory, University of Oxford, England, UK) [33] and manual editing when necessary. The prepared images were then automatically segmented into tissue classes–total GM (cortical + subcortical), total WM and cerebrospinal fluid (CSF) using unified segmentation [34] (Fig 2). This was carried out using SPM8 software (Wellcome Department, University College, London, UK) running on MATLAB version 7.5 (MathWorks, Natrick, MA, USA). All tissue images were visually inspected for accuracy. The template-O-Matic (TOM) toolbox was used to create custom probability maps [35]. This approach uses the US National Institute of Health’s (NIH) MRI Study of Normal Brain Development and general linear models to assess how demographic variables affect the brain structures of 404 children aged 5–18 [35]. Custom tissue probability maps can then be created by matching these regression parameters to the demographics of a pediatric population of interest, and demographic variables of our population (age, gender) were provided to accordingly create a fitting average template from its database [35]. The segmented brain tissues were spatially normalized by using DARTEL [36]. The images were then modulated and smoothed with a 6 mm- Gaussian kernel at full with half maximum. The Easy_Volume toolbox was used to calculate volumes in cm^3^ at 10 years of age for WM, GM, CSF, cerebral parenchyma (CPAR)—defined as GM plus WM, and intracranial volume (ICV)—defined as GM plus WM plus CSF [37].

### Statistical analysis

All the data were tested for normality and homogeneity before the analyses. In order to compare the EPT infants with the control infants and the EPT children with the control children for unadjusted analyses we used the two-sample, between-subjects Student’s *t-*test for the normally distributed continuous variables and the Mann-Whitney *U* test for the non-normally distributed continuous variables. Group differences for the categorical variables were examined with the Fisher’s exact test or the Pearson’s chi-squared test. For adjusted analyses the brain volumes were compared using linear regression, fitted using generalized estimating equation with robust estimation of standard errors to allow for any correlations for multiple births within a family. Cross-sectional analyses with separate models for each brain tissue and structure, and at each time point (term age and 10 years of age) were used. The analyses for the brain tissues WM, GM and CSF were adjusted for sex and ICV, while CPAR and ICV were adjusted for sex. Results are presented as means and mean differences of absolute brain volumes in cubic centimetres (cm^3^) between preterm infants/children and control infants/children. Statistical analyses were performed using SPSS, version 25 (IBM corp, Armonk, NY, USA). Intracranial volume was added as a covariate when comparing brain volumes to adjust for the scaling effect of the brain [38, 39]. Brain volumes are also presented as relative brain volumes—GM or WM as percentages of ICV, adjusted for sex. To account for multiple comparisons, the Benjamini-Hochberg procedure [40] was used for analyses of brain volumes, *Q* was set to 0.2, and m to the number of brain tissues. The effect sizes for the adjusted group differences were calculated using Cohen *d* [41], where 0.2 was considered a small effect size, 0.5 a medium effect size and 0.8 a large effect size [41].

A statistical significance level of p<0.05 was used in all analyses.

## Results

### Study population

The characteristics for the included EPT and control infants and children are summarized in Table 1 and the perinatal characteristics for the included EPT infants and children are summarized in S1 Table.

**Table 1:** Characteristics for included EPT and control infants and children.

	**EPT infants with MRI at term age n = 45**	**Control infants with MRI at term age n = 15**	***p*-value**
Gestational age, median (range) weeks	25.6 (23.3–26.6)	38.9 (38.4–39.9)	^b^<0.001
Birth weight, mean (SD)	829 (160)	3713 (303)	^a^<0.001
Gestational age at MRI, median (range) weeks	40.7 (39.1–45.3)	40.4 (39.9–41.9)	^b^0.24
Sex male n (%)	25 (56)	7 (47)	0.55
	**EPT children with MRI at 10 years n = 51**	**Control children with MRI at 10 years n = 38**	***p*-value**
Gestational age, median (range) weeks	25.6 (23.6–26.6)	40.1 (37.3–41.6)	^b^<0.001
Birth weight, mean (SD)	846 (148)	3739 (454)	^a^<0.001
Age at MRI, median (range) years	10.3 (9.0–11.8)	10.1 (8.3–11.6)	^b^0.45
Sex male n (%)	24 (47)	19 (50)	0.78

MRI = magnetic resonance imaging, SD = standard deviation.

^a^Student’s t test

^b^Mann-Whitney U.

Drop-out analyses showed that the majority of perinatal characteristics were similar for the included EPT infants and children and the EPT infants and children that either declined participation or had low quality on MRI at term age or at 10 years of age (S1 Table). The exceptions were that GA was higher and sepsis and bronchopulmonary dysplasia were less common for the included EPT children at 10 years of age.

The majority of the perinatal characteristics for the 27 EPT children who were included at both time points were similar to the EPT infants or children that were included at only one time point (S2 Table). At term age the 27 EPT infants included at both time points had higher GA, and patent ductus arteriosus ligation, intraventricular haemorrhage grade 1 and 2 and bronchopulmonary dysplasia were less common than for the EPT infants included only at term age. At 10 years of age the 27 EPT children included at both time points had lower incidence of retinopathy of prematurity and bronchopulmonary dysplasia than the EPT children included at only 10 years of age (S2 Table).

The anthropometric measurements at 12 years of age are summarized in S3 Table. At 12 years of age, the EPT children tended to be shorter and have a smaller head circumference compared to the control children, but there were no statistically significant differences in height, weight, Z-score for height, Z-score for weight, BMI or head circumference.

### Brain volumes

#### Grey and white matter brain volumes at term age

The EPT infants had significantly smaller GM volume compared to the control infants when the results were adjusted for ICV and sex. There was no significant difference in adjusted WM volume (Table 2). The significant results remained after correcting for multiple comparisons. For the 27 EPT infants included at both time points there was also smaller adjusted GM volume (S4 Table).

**Table 2:** Brain volumes at term age and at 10 years of age for included extremely preterm (EPT) infants and children, compared to control infants and children.

**Term age**	**EPT infants**	**Control infants**	**Mean difference**	***p-*value**
	**n = 45**	**n = 15**	**(95% CI)**	
GM, mean (SD) cm^3^	200.7 (5.4)	205.7 (6.1)	−5.0 (−8.4, −1.5)	^**a**^ **0.004**
WM, mean (SD) cm^3^	148.2 (4.6)	149.0 (4.4)	−0.9 (−3.5, 1.7)	^a^0.50
CSF, mean (SD) cm^3^	85.8 (9.4)	80.1 (10.4)	5.7 (−0.2, 11.6)	^a^0.058
CPAR, mean (SD) cm^3^	347.4 (39.6)	357.6 (24.0)	−10.1 (−26.9, 6.7)	^b^0.24
ICV, mean (SD) cm^3^	433.3 (40.2)	437.7 (22.5)	−4.4 (−20.0, 12.0)	^b^0.60
**10 years of age**	**EPT children**	**Control children**	**Mean difference**	***p-*value**
	**n = 51**	**n = 38**	**(95% CI)**	
GM, mean (SD) cm^3^	760.8 (11.4)	755.1 (12.6)	5.6 (0.6, 10.6)	^**a**^ **0.028**
WM, mean (SD) cm^3^	456.1 (13.3)	462.1 (10.6)	−6.0 (−10.9, −1.0)	^**a**^ **0.010**
CSF, median (range) cm^3^	200.0 (12.2)	199.6 (14.2)	0.4 (−4.6, 5.3)	^a^0.88
CPAR, mean (SD) cm^3^	1198.3 (84.5)	1244.5 (104.2)	−46.2 (−86.4, −6.0)	^**b**^ **0.024**
ICV, mean (SD) cm^3^	1394.9 (97.8)	1449.2 (123.1)	−54.4 (−101.7, −7.1)	^**b**^ **0.024**

MRI = magnetic resonance imaging, GM = grey matter, WM = white matter, ICV = intracranial volume, CPAR = cerebral parenchyma, CSF = cerebrospinal fluid.

^a^Generalized estimating equations, adjusted for ICV + sex.

^b^Generalized estimating equations, adjusted for sex. Bold values remained significant after correcting for multiple comparisons using the Benjamini-Hochberg procedure.

#### Grey and white matter brain volumes at 10 years of age

At 10 years of age the WM volume was significantly smaller for the EPT children when adjusted for ICV and sex. However, the adjusted GM volume was larger for the EPT children than the control children (Table 2). The significant results remained after correcting for multiple comparisons. The results for the 27 children included at both time points compared to the control children also found smaller adjusted WM volume, but the adjusted GM volume was not significantly larger (S4 Table).

In S2 Table the brain volumes for the 27 EPT children that were included at both time points were compared to the EPT infants (n = 18) and EPT children (n = 24) included at one time point, and there were no differences in GM or WM volumes.

#### Differences in intracranial volume, cerebral parenchyma and cerebrospinal fluid at term age and at 10 years of age

At term age there were no significant differences in CPAR or ICV, but at 10 years of age CPAR and ICV were significantly smaller (Table 2). The adjusted CSF volumes were not different between the EPT children and control children at term age or at 10 years of age.

All unadjusted brain volumes are found in S4 and S5 Tables.

#### Effect sizes and relative volumes for white and grey matter brain volumes

**Fig 3:**
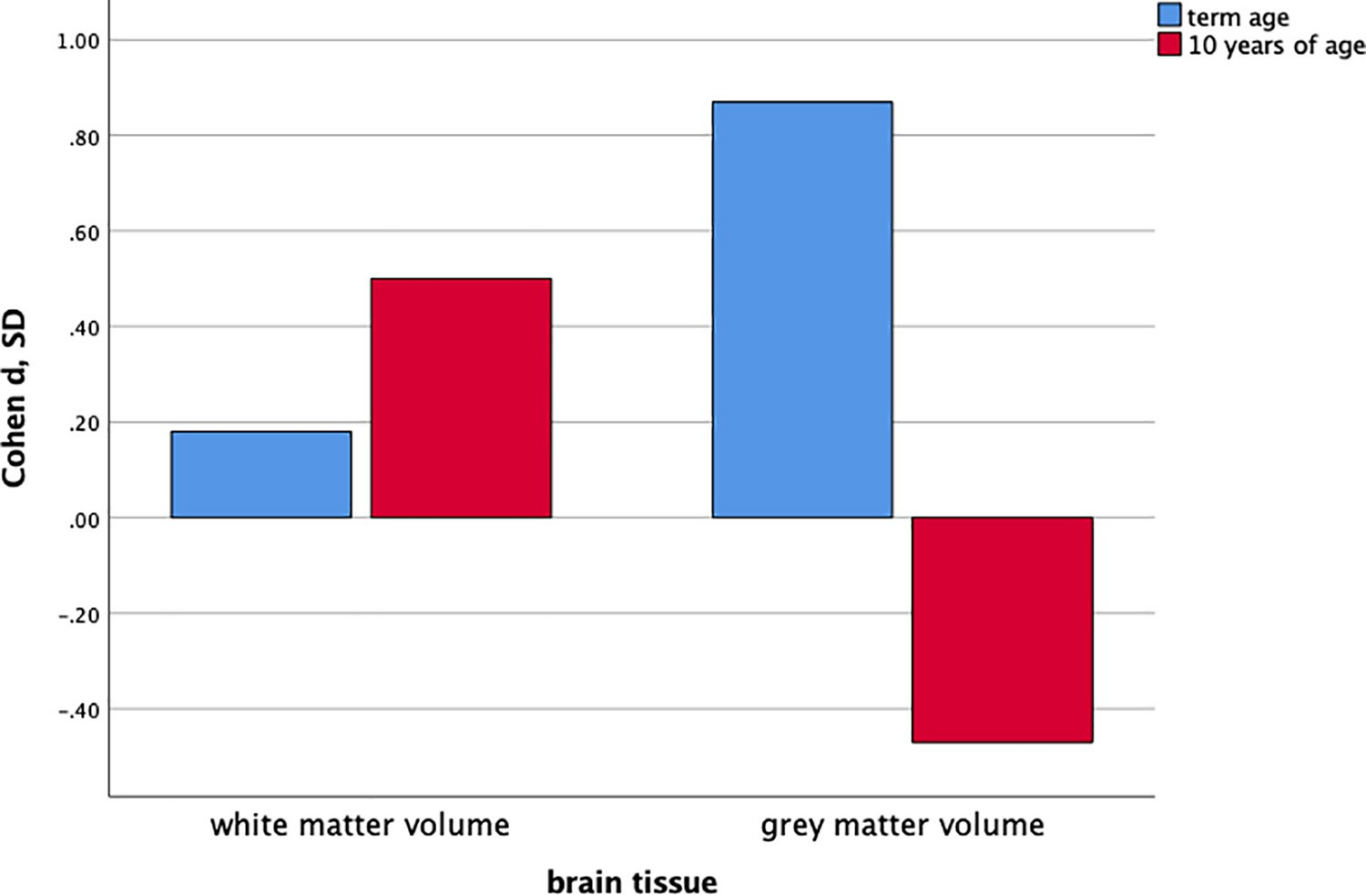
Effect size calculations for brain volumes at term age and at 10 years of age. Effect size (Cohen *d*, SD) calculations at term age (45 extremely preterm infants and 15 control infants) and at 10 years of age (51 extremely preterm children and 38 control children) for white and grey matter volumes.

Effect sizes calculated for the adjusted group differences demonstrated a large effect size for GM volume, and a small effect size for WM volume at term age. At 10 years of age the effect size for reduced WM volume, and the effect size for larger GM volume for EPT children were moderate (Fig 3). The relative volumes are presented as GM and WM as percentage of ICV adjusted for sex in S6 Table. The relative GM volume was significantly reduced at term age for the EPT infants compared to the control infants. At 10 years of age the relative WM volume was significantly reduced for the EPT children compared to the control children, and relative GM volume was significantly larger for the EPT children compared to the control children, therefore the results for the relative volumes match the results for the absolute volumes.

## Discussion

We investigated the volumes of white and grey matter at term age and at around 10 years of age in a well-defined cohort of children born EPT, up to GA 26 weeks and 6 days, compared to term born controls. To our knowledge, this study is the first to describe WM and GM volumes at both term age and 10 years of age in cohort consisting of only EPT children. The main findings of this study were that the EPT infants had smaller adjusted GM volume at term age and the EPT children had smaller adjusted WM volume at 10 years of age compared with full term controls. The differences in cerebral parenchyma and intracranial volume between EPT children and controls were more prominent at 10 years of age than at term age, and no difference was found for CSF volume.

### Grey and white matter volumes at term age

At term age, the GM volume was smaller for the EPT infants compared to the control infants. We found no difference for WM volume between EPT infants and control infants. Brain growth during the third trimester of pregnancy is rapid in normal pregnancies and after 33 weeks there is a proportional increase in GM volume compared to ICV in the fetus [42]. Children born EPT are exposed to an early ex-utero environment with altered sensory inputs due to parental absence, procedures, noise and light. Altered sensory inputs affects the normal brain maturation [43]. These children also experienced nutritional challenges during early critical windows of brain development [44]. A combination of these factors could be affecting the critical period of synaptogenesis and leading to atypical trajectories of brain growth resulting in reduced GM volume [42].

A majority of previously published studies have reported that differences in GM volume between preterm infants and control infants were most prominent at term age [12, 45, 46]. However, there are previous studies that have reported contradictory findings regarding whether WM volume is smaller for preterm infants than for control infants at term age [11, 20]. These discrepancies could be partly explained by differences in the inclusion criteria and study methods, for example including children with different gestational ages and infants who had complications related to preterm birth such as intraventricular haemorrhage, severe white matter injuries or periventricular leukomalacia.

### Grey and white matter brain volumes at 10 years of age

At 10 years of age we found smaller WM and larger GM volumes for the EPT children compared to the control children. The interplay between WM and GM during normal brain development is still not fully understood [47–49]. It is reported from previous research that by six years of age term born children already have about 90% of the adult ICV [17, 47–50]. Recent knowledge about normal brain development entails a decrease in GM volume relative to ICV throughout childhood and cortical thinning beginning at around 3 years of age [48, 50–52]. In contrast, WM volume increases during the entire childhood and up to at least the fourth decade of life [48, 50, 53–55]. This reflects the maturation and myelination of organized brain connections that increase during childhood, linked to increased WM volume [48, 56]. Both dendritic pruning and myelination in the interface between cortical GM and WM have been linked to cortical thinning and a reduction of GM volume over time [47, 48, 50, 51, 56]. An early environmental risk, such as the preterm extra uterine environment, could lead to alternative brain trajectories [6, 57]. These could include possible disturbances of normal brain maturation, with expected increases in WM volume and decreases in GM volume [6, 29, 57–59].

Our finding that the adjusted WM volume was smaller for the EPT children than for the control children at 10 year of age is in line with previous reports. One previous study reported that preterm children with a weight of less than 1250 g had less increase of WM and less decrease of GM from age 8 to 12 years compared to term born controls [60]. Thus, the expected normal development, with a WM increase and a GM decrease, was less marked in preterm children compared to controls. Another cross-sectional study reported significantly smaller WM volume at the age of 16 years in children born before GA 32 weeks compared to term born controls [24].

We also found that the GM volume was larger in the EPT children at 10 years of age and smaller at term age compared to control children which could possibly be due to a disrupted cortical thinning as a result of extreme prematurity. Disrupted cortical thinning has previously been seen in low birth weight children compared to control children [61]. We expect that the found differences in brain volumes are not only due to a delayed maturation, but also due to stable changes not compensated over time.

There are studies comparing children born very preterm with full term controls during childhood and adolescence that have found either that GM volume was more reduced than WM volume in the preterm children or an equal effect on GM and WM volumes [20, 62]. A longitudinal study that compared very preterm children at less than 30 weeks of gestation or weighing less than 1250 g with full term children showed less growth in both GM and WM volumes from term age to 7 years of age, with a dominance in growth reduction in GM volume [20]. That the present study found smaller WM volume but larger GM volume at 10 years of age, might be partly explained by the different age at the follow-up MRI scan, the exclusion of children with intraventricular haemorrhage grade 3–4, severe white matter injuries or periventricular leukomalacia and the lower GA of our cohort. A number of studies have reported relationships between brain volumes and gestational age [18, 21, 46, 63], but they did not include many children born EPT. Lower gestational age has been reported to have a greater effect on WM volume [18, 63].

More advanced techniques have been applied to investigate the white matter integrity in preterm children. Fixel based analysis found lower fibre density and fibre cross-section in very preterm children compared to full term controls at age 7 and 13 years, and the differences were larger with lower GA [64]. Diffusion tensor imaging (DTI) and neurite orientation and dispersion density imaging (NODDI) have found lower fractional anisotropy and higher neurite orientation dispersion index in very preterm children compared to term born controls [65]. These previous findings lead us to speculate that the smaller WM volume for the EPT children found in this study is reflecting altered underlying microstructural properties of WM.

### Differences in intracranial volume, cerebral parenchyma and cerebrospinal fluid at term age and at 10 years of age

We found that CPAR and ICV were significantly smaller for the EPT children compared to the controls at 10 years of age, but this was not the case at term age. CSF volume was not different between EPT children and controls. Also previous studies have reported similar ICV between children <1000 grams and control children at term age [13]. The smaller ICV at 10 years of age is in line with previous cross-sectional studies, which demonstrated smaller ICV in very preterm [24] and EPT children [23] compared with controls in childhood and adolescence. Also, a longitudinal study focusing on regional brain growth of children born before 30 weeks of gestation demonstrated that ICV remained smaller in preterm children than in term born controls at age 7 and 13 years of age [16]. Studies that investigated brain volumes in adults who were born very preterm have demonstrated that the small overall brain volumes seen in preterm children persisted into adulthood [17, 19]. Thus, even though the survival rates and care of the children with the lowest GA are developing we still see smaller ICV and CPAR in relatively healthy EPT children without major morbidities compared to control children up to 10 years of age. The brain parenchyma is affected, since we found no difference in CSF volume. We found no significant difference in anthropometric measurements at age 12. Even though this tells us little about their body size at the time when the children were imaged it is an indication that our cohort is relatively healthy, and even so we found significant brain tissue reduction at 10 years of age. It is possible that the mean differences in brain volumes that we found between EPT children and controls might be clinically relevant, since previous studies have reported that similar altered brain volumes in preterm children were accompanied by adverse cognitive outcomes [18, 20, 24]. Studies on cognition, motor and behavioural outcomes at 12 years of age in our cohort are ongoing.

### Strengths and limitations

A strength was that this was a population-based study that focused exclusively on EPT children up to GA 26 weeks and 6 days. Because we wanted to disentangle the effect of extreme prematurity on brain volumes, we excluded focal brain lesions, congenital conditions and many complications related to preterm birth. We carefully selected high quality scans for the volumetric analyses. Another strength of the study was the long follow-up period including two MRI scans with 10 years apart.

A limitation of the study was the relatively small sample sizes that may have prevented statistical differences to be discerned. This was due to rigorous data quality criteria and implicit methodological difficulties. Furthermore, we only included children without major morbidities on their conventional MRI scans, in order to reflect the process of normal brain development. Another limitation of the study was the use of two separate control groups at term age and at 10 years of age, however the two control groups had similar perinatal characteristics and the brain volumes were comparable to previously published brain volumes of healthy term born children at both time points [13, 66].

The MRI scans at term age and at 10 years of age were performed with different MRI acquisition parameters and on different scanners with different field strengths. This was unavoidable, as the original scanner was no longer available when the children reached the age of 10 years. However, previous studies indicate that brain volumes can be robustly measured across different scanners, field strengths and acquisitions [67, 68]. But the aggregation of neuroimaging data across scanners could potentially increase statistical power to detect biological variability of interest. Additionally, the default processing pipelines can be customized to increase accuracy of segmentation and normalization, yet the impact of customizations on analyses in EPT children are not clear. Extremely preterm populations have specific brain anatomical features that prevented us from creating customized templates [69]. Although we used the default DARTEL pipeline the brain volumes reported here were in line with those reported in previously published studies including similar populations [20, 66].

## Conclusion

In this MRI study of EPT children, the largest differences in adjusted GM and WM volumes between EPT children and controls were in GM at term age and in WM at 10 years of age. In addition, ICV and CPAR were smaller for the EPT children at 10 years of age compared to the term born controls, but this was not the case at term age, and no difference was found for CSF volume at either time point. This suggest that EPT birth results in long-lasting effects on brain volumes for this vulnerable group, with a different pattern of GM and WM distribution. Our findings highlight the need for long-term follow-up studies that focus on the outcomes of being born EPT, including cognitive data.

## Supporting information

S1 Fig Flow chart for control infants and children. Full term control infants and children who underwent magnetic resonance imaging (MRI) at term age and 10 years of age.
(TIFF)

S1 Table Drop-out analyses for EPT infants and children not included due to declined participation or low quality on MRI and finally included EPT infants and children. (DOCX)

S2 Table Characteristics and brain volumes for the EPT infants and children included at both term age and 10 years of age (n = 27) compared to the EPT infants and children included at only one time point. (DOCX)

S3 Table Anthropometric measurements of the extremely preterm (EPT) children and the control children with high quality MRI data at 10 years of age with available clinical follow-up data at 12 years of age. (DOCX)

S4 Table Brain volumes at term age and 10 years of age for extremely preterm (EPT) infants and children with high quality MRI data at both term age and 10 years of age, compared to controls. (DOCX)

S5 Table Unadjusted brain volumes at term age and at 10 years of age for included extremely preterm (EPT) infants and children, compared to control infants and children. (DOCX)

S6 Table Grey and white matter volumes relative to intracranial volume (ICV) at term age and 10 years of age, adjusted for sex. (DOCX)

S1 File (XLSX)
