# Supplementary figures and images for: Exploring the distribution of grey and white matter brain volumes in extremely preterm children, using magnetic resonance imaging at term age and at 10 years of age
Source: PLoS One. 2021 Nov 5;16(11):e0259717. doi: 10.1371/journal.pone.0259717 (PMC8570467; doi:10.1371/journal.pone.0259717)

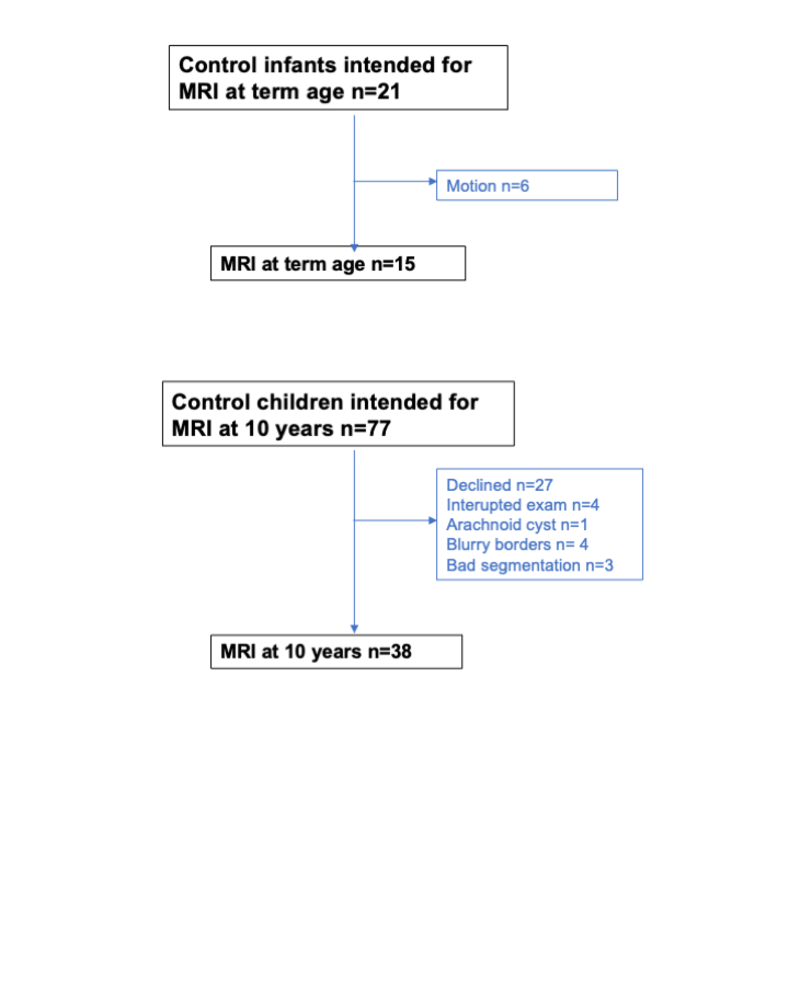

Supplement: S1 Fig — Full term control infants and children who underwent magnetic resonance imaging (MRI) at term age and 10 years of age. (TIFF) [file pone.0259717.s001.tiff]
