# Supplementary material for: Exploring the distribution of grey and white matter brain volumes in extremely preterm children, using magnetic resonance imaging at term age and at 10 years of age
Source: PLoS One. 2021 Nov 5;16(11):e0259717. doi: 10.1371/journal.pone.0259717 (PMC8570467; doi:10.1371/journal.pone.0259717)
Supplement: S1 Table — (DOCX) [file pone.0259717.s002.docx]

**S1 Table.**

**a)** Drop-out analyses for EPT infants not included due to declined participation or low quality on MRI and finally included EPT infants (n=45+62=107).

**b)** Drop-out analyses of EPT children not included due to declined participation or low quality on MRI and finally included EPT children (n=51+41=92).

|  |  | **a)** |  |  | **b)** |  |
| --- | --- | --- | --- | --- | --- | --- |
|  | **EPT infants included**  **n=45** | **EPT infants**  **not included**  **n=62** | ***p*-value** | **EPT children included**  **n=51** | **EPT children not included**  **n=41** |  |
| Gestational age, median  (range) weeks | 25.6  (23.3-26.6) | 25.5  (23.4-26.8) | ^b^0.37 | 25.6  (23.6-26.6) | 25.1  (23.3-26.6) | ^b^0.024 |
| Birth weight, mean (SD), g | 827 (161) | 808 (166) | ^a^0.54 | 846 (148) | 791 (172) | ^a^0.11 |
| Sex male, n (%) | 25 (56) | 37 (60) | 0.67 | 24 (47) | 25 (61) | 0.18 |
| Antenatal steroids, n (%) | 43 (96) | 57 (92) | 0.70 | 48 (94) | 38 (93) | 1.0 |
| Patent ductus arteriosus, n (%) | 33 (73) | 44 (71) | 0.79 | 36 (71) | 31 (76) | 0.59 |
| Patent ductus arteriosus ligation, n (%) | 14 (31) | 18 (29) | 0.82 | 16 (31) | 14 (34) | 0.78 |
| Patent ductus arteriosus medically treated, n (%) | 33 (73) | 41 (66) | 0.43 | 34 (67) | 30 (73) | 0.50 |
| Multiple births, n (%) | 10 (22) | 9 (15) | 0.16 | 9 (18) | 7 (17) | 0.72 |
| Sepsis, n (%) | 33 (73) | 49 (79) | 0.49 | 36 (71) | 37 (90) | 0.021 |
| Intraventricular haemorrhage grade 1-2, n (%) | 18 (40) | 21 (34) | 0.52 | 16 (31) | 18 (43) | 0.22 |
| Small for gestational age <2SD, n | 3 (7) | 9 (15) | 0.23 | 4 (8) | 5/36 (12) | 0.51 |
| Retinopathy of prematurity stage ≥3, n (%) | 13/45 (37) | 27/54 (50) | 0.23 | 18/39 (46) | 15 (42) | 0.24 |
| Necrotizing enterocolitis, n (%) | 4 (9) | 11 (18) | 0.26 | 7 (14) | 7 (17) | 0.66 |
| WM abnormalities, none, n (%)  mild, n (%)  moderate, n | 24 (53)  20 (44)  1 (2) | 28/52 (55)  21/52 (41)  2/52 (3) | 0.99 | 26 (54)  21 (44)  1 (2) | 17/48 (45)  18/48 (47)  3/48 (6) | 0.23 |
| Bronchopulmonary dysplasia, n (%) | 16 (36) | 28/57 (49) | 0.17 | 18/50 (36) | 24/40 (60) | 0.023 |

MRI=magnetic resonance imaging, SD=standard deviation, WM=white matter, ^a^Student’s t test, ^b^Mann-Whitney U
