# Supplementary material for: Exploring the distribution of grey and white matter brain volumes in extremely preterm children, using magnetic resonance imaging at term age and at 10 years of age
Source: PLoS One. 2021 Nov 5;16(11):e0259717. doi: 10.1371/journal.pone.0259717 (PMC8570467; doi:10.1371/journal.pone.0259717)
Supplement: S2 Table — (DOCX) [file pone.0259717.s003.docx]

|  |  | **a)** |  | **b)** | | |
| --- | --- | --- | --- | --- | --- | --- |
|  | **EPT children included at both time points**  **n=27** | **EPT infants included at only term age**  **n=18** | ***p*-value** | **EPT children included at both time points**  **n=27** | **EPT children included at only 10 years of age**  **n=24** | ***p-*value** |
| Gestational age, median  (range) weeks | 26.3  (24.3-26.6) | 25.1  (23.3-26.4) | ^b^0.005 | 26.3  (24.3-26.6) | 25.6 (23.6-26.6) | ^b^0.051 |
| Birth weight, mean (SD), g | 863 (138) | 773 (181) | ^a^0.067 | 863 (138) | 827 (160) | ^a^0.39 |
| Sex male, n (%) | 13 (48) | 12 (67) | 0.36 | 13 (48) | 11 (46) | 0.87 |
| Antenatal steroids, n (%) | 26 (96) | 17 (94) | 1.0 | 26 (96) | 22 (92) | 0.48 |
| Patent ductus arteriosus, n (%) | 18 (67) | 15 (83) | 0.31 | 18 (67) | 18 (75) | 0.55 |
| Patent ductus arteriosus ligation, n (%) | 4 (15) | 10 (56) | 0.007 | 4 (15) | 12 (50) | 0.14 |
| Patent ductus arteriosus medically treated, n (%) | 18 (67) | 15 (83) | 0.31 | 18 (67) | 16 (67) | 0.99 |
| Multiple births, n (%) | 5 (19) | 5 (28) | 0.59 | 5 (19) | 4 (17) | 0.52 |
| Sepsis, n (%) | 18 (67) | 15 (83) | 0.31 | 18 (67) | 18 (75) | 0.51 |
| Intraventricular haemorrhage grade 1-2, n (%) | 7 (26) | 11 (61) | 0.018 | 7 (26) | 9 (38) | 0.37 |
| Small for gestational age <2SD, n | 2 (7) | 1 (6) | 1.0 | 2 (7) | 2 (8) | 1.0 |
| Retinopathy of prematurity stage ≥3, n (%) | 5/18 (28) | 8/17 (47) | 0.24 | 5/18 (28) | 13/21 (35) | 0.033 |
| Necrotizing enterocolitis, n (%) | 2 (7) | 2 (11) | 0.39 | 2 (7) | 5 (21) | 0.31 |
| WM abnormalities, none, n (%)  mild, n (%)  moderate, n | 13 (48)  13 (48)  1 (4) | 11 (61)  7 (39)  0 (0) | 0.32 | 13 (48)  13 (48)  1 (4) | 13/21 (62)  8/21 (38)  0/21 (0) | 0.27 |
| Bronchopulmonary dysplasia, n (%) | 5 (36) | 11 (60) | 0.003 | 5 (19) | 13/23 (57) | 0.005 |
| Grey matter volume adjusted for ICV | 201.0 (5.9) | 199.2 (5.9) | ^c^0.32 | 746.7 (10.9) | 750.1 (10.8) | ^c^0.27 |
| White matter volume adjusted for ICV | 148.5 (5.0) | 146.9 (5.1) | ^c^0.33 | 448.0 (13.0) | 448.2 (13.0) | ^c^0.97 |

**S2 Table. a)** Characteristics and brain volumes for the EPT children included at both term age and 10 years of age (n=27) compared to the EPT children included at only term age (n=18).

**b)** Characteristics and brain volumes for the EPT children included at both term age and 10 years of age (n=27) compared to the EPT children included at only 10 years of age (n=24).

MRI=magnetic resonance imaging, SD=standard deviation, WM=white matter, ^a^Student’s t test, ^b^Mann-Whitney U, ^c^Ancova adjusted for ICV
