# Supplementary material for: Exploring the distribution of grey and white matter brain volumes in extremely preterm children, using magnetic resonance imaging at term age and at 10 years of age
Source: PLoS One. 2021 Nov 5;16(11):e0259717. doi: 10.1371/journal.pone.0259717 (PMC8570467; doi:10.1371/journal.pone.0259717)
Supplement: S3 Table — (DOCX) [file pone.0259717.s004.docx]

|  | **EPT children at 12 years** | **Controls at 12 years** | ***p-* value** |
| --- | --- | --- | --- |
| Age at clinical follow-up, n, mean (SD), years | 34/51, 12.1 (0.3) | 25/38, 12.0 (0.2) | ^a^0.12 |
| Weight, n, median  (range), kilograms | 34/51, 41.4 (27.4-72.9) | 25/38, 41.0 (33.4-63.6) | ^b^0.86 |
| Weight Z-score, n, median  (range) | 34/51, -0.12 (-2.0-4.2) | 25/38, -0.15 (-1.1-3.0) | ^b^0.74 |
| Height, n, mean (SD), cm | 33/51, 151.2 (8.2) | 23/38, 154.8 (5.9) | ^a^0.077 |
| Height Z-score, n, mean (SD) | 33/51, -0.29 (1.05) | 23/38, 0.15 (0.79) | ^a^0.092 |
| BMI, n, median (range) | 33/51, 17.5 (14.4-25.7) | 23/51, 17.6 (14.6-24.5) | ^b^0.43 |
| Head circumference, n, mean (SD), cm | 30/51, 53.3 (2.6) | 16/38, 54.3 (1.2) | ^a^0.15 |

**S3 Table: Anthropometric measurements for the extremely preterm (EPT) children and the control children with high quality MRI data at 10 years of age with available clinical follow-up data at 12 years of age.**

EPT= extremely preterm children, SD=standard deviations

^a^Student’s t test, ^b^Mann-Whitney U
