# Supplementary material for: Exploring the distribution of grey and white matter brain volumes in extremely preterm children, using magnetic resonance imaging at term age and at 10 years of age
Source: PLoS One. 2021 Nov 5;16(11):e0259717. doi: 10.1371/journal.pone.0259717 (PMC8570467; doi:10.1371/journal.pone.0259717)
Supplement: S4 Table — (DOCX) [file pone.0259717.s005.docx]

| **Term age** | **EPT infants, unadjusted**  **n=27** | **Control infants, unadjusted n=15** | **Mean difference, unadjusted**  **(95% CI)** | ***p-*value** | **EPT infants, adjusted***  **n=27** | **Control infants,**  **adjusted***  **n=15** | **Mean difference, adjusted***  **(95% CI)** | ***p-*value** |
| --- | --- | --- | --- | --- | --- | --- | --- | --- |
| GM, mean (SD) cm^3^ | 207.8  (24.6) | 207.1  (13.6) | 0.7  (−13.2, 14.6) | ^a^0.92 | 206.0  (4.2) | 210.3  (5.8) | −4.3  (−7.8, −0.9) | **^c^0.014** |
| WM, mean  (SD) cm^3^ | 153.6  (18.7) | 150.1  (10.1) | 3.5  (−7.0, 14.1) | ^a^0.51 | 152.2  (4.4) | 152.5  (4.3) | −0.2  (−3.0, 2.5) | ^c^0.87 |
| CSF, mean  (SD) cm^3^ | 84.8  (7.4) | 80.2  (10.4) | 4.7  (−0.9, 10.3) | ^a^0.096 | 84.8  (7.2) | 80.3  (10.1) | 4.5  (−1.4, 10.5) | ^c^0.13 |
| CPAR, mean (SD) cm^3^ | 361.4  (43.0) | 357.1  (23.6) | 4.2  (−12.0, 20.1) | ^a^0.73 | 361.8 (37.4) | 358.0  (26.7) | 3.8  (−15.7, 23.4) | ^d^0.70 |
| ICV, mean  (SD) cm^3^ | 446.2  (42.7) | 437.3  (23.2) | 8.9  (−15.2, 33.1) | ^a^0.46 | 446.7 (37.4) | 438.2  (24.3) | (−10.3, 27.4) | ^d^0.38 |
| **Late childhood** | **EPT children**  **unadjusted**  **n=27** | **Control children,**  **unadjusted**  **n=38** | **Mean difference, unadjusted**  **(95% CI)** | ***p-*value** | **EPT children adjusted***  **n=27** | **Control children,**  **adjusted***  **n=38** | **Mean difference, adjusted***  **(95% CI)** | ***p-*value*** |
| GM, mean  (SD) cm^3^ | 745.6  (60.4) | 771.8  (63.9) | −26.3  (−57.7, 5.1) | ^a^0.10 | 763.2  (9.8) | 759.4  (12.5) | 3.6  (−1.6, 8.9) | ^c^0.18 |
| WM, mean  (SD) cm^3^ | 447.3  (42.2) | 472.7  (40.1) | −25.4  (−46.3, −4.4) | **^a^0.014** | 458.7  (14.0) | 464.6  (10.5) | −5.9  (−11.6, −0.2) | ^c^**0.032** |
| CSF, median  (range) cm^3^ | 196.0  (168.4-255.4) | 199.0  (169.1-338.4) | - | ^b^0.30 | 203.1 (11.4) | 200.8 (14.2) | −2.3  (−2.8, 7.4) | ^c^0.38 |
| CPAR, mean  (SD) cm^3^ | 1192.9  (100.1) | 1244.5  (103.2) | −51.7  (−102.9, −0.4) | **^a^0.048** | 1194.0  (82.6) | 1244.5  (103.6) | −50.5  (−95.7, −5.3) | **^d^0.028** |
| ICV, mean  (SD) cm^3^ | 1390.5  (116.3) | 1449.2  (121.1) | −58.8  (−118.6, 1.2) | ^a^0.055 | 1391.8  (95.6) | 1449.4  (122.7) | −57.4  (−110.4, 4.5) | **^d^0.033** |

**S4 Table: Brain volumes at term age and 10 years of age for extremely preterm (EPT) infants and children with high quality MRI data at both term age and 10 years of age, compared to controls.**

MRI=magnetic resonance imaging, CSF=cerebrospinal fluid, GM=grey matter, WM=white matter, CPAR=cerebral parenchyma, ICV=intracranial volume.

^a^Student’s t test, ^b^Mann-Whitney U, ^c^Generalized estimating equations adjusted for sex and ICV, ^d^Generalized estimating equations adjusted for sex. Bold values remained significant after correcting for multiple comparisons using the Benjamini-Hochberg procedure.
