# Supplementary material for: Exploring the distribution of grey and white matter brain volumes in extremely preterm children, using magnetic resonance imaging at term age and at 10 years of age
Source: PLoS One. 2021 Nov 5;16(11):e0259717. doi: 10.1371/journal.pone.0259717 (PMC8570467; doi:10.1371/journal.pone.0259717)
Supplement: S5 Table — (DOCX) [file pone.0259717.s006.docx]

**S5 Table. Unadjusted brain volumes at term age and at 10 years of age for the included extremely preterm (EPT) infants and children, compared to control infants and children.**

| **Term age** | **EPT infants unadjusted**  **n=45** | **Control infants, unadjusted n=15** | **Mean difference, unadjusted**  **(95% CI)** | ***p-*value** |
| --- | --- | --- | --- | --- |
| GM, mean (SD) cm^3^ | 200.3 (23.6) | 207.1 (13.6) | −6.7 (−19.7, 6.1) | ^a^0.30 |
| WM, mean (SD) cm^3^ | 147.9 (17.9) | 150.1 (10.1) | −2.2 (−12.0, 7.6) | ^a^0.65 |
| CSF, mean (SD) cm^3^ | 85.7 (9.7) | 80.2 (10.4) | 5.7 (−0.3, 11.4) | ^a^0.063 |
| CPAR, mean (SD) cm^3^ | 348.2 (41.4) | 357.1 (23.6) | −9.0 (−31.6, 13.6) | ^a^0.43 |
| ICV, mean (SD) cm^3^ | 433.9 (44.4) | 437.3 (23.2) | −3.4 (−25.9, 19.1) | ^a^0.76 |
| **10 years of age** | **EPT children**  **unadjusted**  **n=51** | **Control children,**  **unadjusted**  **n=38** | **Mean difference, unadjusted**  **(95% CI)** | ***p-*value** |
| GM, mean (SD) cm^3^ | 748.3 (60.2) | 771.8 (63.9) | −23.5 (−49.8, 2.8) | ^a^0.079 |
| WM, mean (SD) cm^3^ | 448.1 (40.2) | 472.7 (41.1) | −24.6 (−41.9, −7.4) | **^a^0.005** |
| CSF, median (range) cm^3^ | 194.7 (152.1-255.4) | 199.0 (169.1-338.4) | - | ^b^0.084 |
| CPAR, mean (SD) cm^3^ | 1196.4 (97.8) | 1244.5 (103.2) | −48.2 (−90.8, −5.5) | **^a^0.027** |
| ICV, mean (SD) cm^3^ | 1392.6 (113.9) | 1449.2 (121.1) | −56.6 (−106.4, −6.2) | **^a^0.027** |

MRI=magnetic resonance imaging, GM=grey matter, WM=white matter, ICV=intracranial volume, CPAR=cerebral parenchyma, CSF=cerebrospinal fluid.

^a^Student’s t test, ^b^Mann-Whitney U. Bold values remained significant after correcting for multiple comparisons using the Benjamini-Hochberg procedure.
