# Supplementary material for: Exploring the distribution of grey and white matter brain volumes in extremely preterm children, using magnetic resonance imaging at term age and at 10 years of age
Source: PLoS One. 2021 Nov 5;16(11):e0259717. doi: 10.1371/journal.pone.0259717 (PMC8570467; doi:10.1371/journal.pone.0259717)
Supplement: S6 Table — (DOCX) [file pone.0259717.s007.docx]

**S6 Table.** **Grey and white matter volumes relative to ICV at term age and 10 years of age, adjusted for sex.**

| **Term age** | **EPT infants**  **n=45** | **Control infants,**  **n=15** | **Mean difference,**  **(95% CI)** | ***p-*value** |
| --- | --- | --- | --- | --- |
| GM, % of ICV  mean (SD) | 46.1 (1.6) | 47.3 (1.4) | −1.3 (−2.1, −0.3) | 0.003^1^ |
| WM, % of ICV  mean (SD) | 34.0 (1.3) | 34.3 (1.1) | 0.28 (−1.0, 0.45) | 0.32^1^ |
| **10 years of age** | **EPT children**  **n=51** | **Control children,**  **n=38** | **Mean difference,**  **(95% CI)** | ***p-*value** |
| GM, % of ICV  mean (SD) | 53.7 (0.8) | 53.3 (1.0) | 0.47 (0.11, 0.84) | 0.012^1^ |
| WM, % of ICV  mean (SD) | 32.2 (0.9) | 32.6 (0.7) | −0.45 (−0.82, −0.09) | 0.012^1^ |

GM=grey matter, WM=white matter, ICV=intracranial volume, ^1^Generalized estimating equations adjusted for sex
